# Sul1 and Sul2 Sulfate Transceptors Signal to Protein Kinase A upon Exit of Sulfur Starvation*

**DOI:** 10.1074/jbc.M114.629022

**Published:** 2015-02-27

**Authors:** Harish Nag Kankipati, Marta Rubio-Texeira, Dries Castermans, George Diallinas, Johan M. Thevelein

**Affiliations:** From the ‡ Laboratory of Molecular Cell Biology, Institute of Botany and Microbiology, KU Leuven, Kasteelpark Arenberg 31, B-3001 Leuven-Heverlee, Flanders, Belgium,; the § Department of Molecular Microbiology, VIB, Kasteelpark Arenberg 31, B-3001 Leuven-Heverlee, Flanders, Belgium, and; the ¶ Faculty of Biology, University of Athens, Panepistimioupolis, Athens 15784, Greece

**Keywords:** Endocytosis, Membrane Transport, Protein Kinase A (PKA), Signaling, Transporter, Symporter, Signaling Agonist, Stationary Phase, Sulfate Sensing, Sulfate Transporter

## Abstract

Sulfate is an essential nutrient with pronounced regulatory effects on cellular metabolism and proliferation. Little is known, however, about how sulfate is sensed by cells. Sul1 and Sul2 are sulfate transporters in the yeast *Saccharomyces cerevisiae*, strongly induced upon sulfur starvation and endocytosed upon the addition of sulfate. We reveal Sul1,2-dependent activation of PKA targets upon sulfate-induced exit from growth arrest after sulfur starvation. We provide two major arguments in favor of Sul1 and Sul2 acting as transceptors for signaling to PKA. First, the sulfate analogue, d-glucosamine 2-sulfate, acted as a non-transported agonist of signaling by Sul1 and Sul2. Second, mutagenesis to Gln of putative H^+^-binding residues, Glu-427 in Sul1 or Glu-443 in Sul2, abolished transport without affecting signaling. Hence, Sul1,2 can function as pure sulfate sensors. Sul1^E427Q^ and Sul2^E443Q^ are also deficient in sulfate-induced endocytosis, which can therefore be uncoupled from signaling. Overall, our data suggest that transceptors can undergo independent conformational changes, each responsible for triggering different downstream processes. The Sul1 and Sul2 transceptors are the first identified plasma membrane sensors for extracellular sulfate. High affinity transporters induced upon starvation for their substrate may generally act as transceptors during exit from starvation.

## Teaser

**Background:** Transceptors are transporters with an additional receptor function.

**Results:** Sul1 and Sul2 signal to PKA in the absence of sulfate transport.

**Conclusion:** Yeast Sul1 and Sul2 are sulfate transceptors.

**Significance:** Transceptors could be a universal class of nutrient sensors.

## Introduction

To address the constant challenge of fluctuating nutrient availability, microorganisms have developed many nutrient-sensing systems, including nutrient sensors located at the plasma membrane (1). Specific transporters with an additional receptor function, designated as transceptors (2), have been identified for extracellular sensing of nutrients (3). So far the Gap1 transceptor has been identified for amino acids (4, 5), Pho84 for phosphate (6, 7), and Mep2 for ammonium (8). They all share the property of being strongly induced by starvation for their substrate. Readdition of the lacking substrate causes transcriptional repression as well as rapid down-regulation of the transceptors by endocytosis (9–13). In addition, upon exit of the cells from the growth arrest, the transceptor mediates nutrient activation of the protein kinase A (PKA) pathway to regulate a range of cellular properties (14). Also in higher eukaryotes, evidence for transporters with an additional nutrient-sensing function has been reported (15–18). However, no plasma membrane sensors for extracellular sulfate have been described in eukaryotic cells, although genetic evidence for a possible regulatory role of the *Arabidopsis* SULTR1;2 sulfate transporter has been obtained (19).

In yeast, sulfur in the form of sulfate is taken up by two high affinity H^+^-symporters, Sul1 and Sul2 (20, 21). They are homologous to each other and orthologous to the fungal SUL, plant SULTR, and animal SLC26 transporter family (22). The sulfate taken up into the cell is reduced to sulfide and homocysteine through the sulfate assimilation pathway and subsequently incorporated into sulfur-containing compounds, like methionine, cysteine, glutathione, and *S*-adenosylmethionine (23). Both Sul1 and Sul2 are strongly induced upon sulfur starvation (24) and endocytosed upon the readdition of sulfate (25), resulting in a rapid drop in sulfate uptake activity (20).

Starvation of yeast cells for an essential nutrient on a glucose medium results in G_1_ arrest, entry into stationary phase, and down-regulation of the PKA pathway (14). This causes accumulation of the reserve and stress protection carbohydrates, glycogen and trehalose (26); acquirement of high stress tolerance; up-regulation of stress response gene expression; and down-regulation of ribosomal protein gene expression (14). Readdition of the nutrient for which the cells were starved in the presence of glucose causes rapid activation of targets of the PKA pathway, like the enzyme trehalase, which increases 5–10-fold in activity upon phosphorylation by PKA and causes mobilization of trehalose. Trehalase activation is a very convenient read-out for rapid nutrient-induced activation of PKA *in vivo* (27–29). Unlike PKA activation by glucose addition to glucose-deprived cells, activation by nitrogen, phosphate, and sulfate in glucose-repressed cells is not associated with an increase in the cAMP level (27, 29). The sulfate sensors responsible for rapid sulfate activation of the PKA pathway in sulfur-starved cells have remained unknown.

We now show that sulfate activation of PKA targets in sulfur-starved cells is dependent on Sul1 and Sul2. We demonstrate that Sul1 and Sul2 act as transceptors by uncoupling their transport and receptor function in two different ways. We also show that sulfate signaling by Sul1 or Sul2 is not required for sulfate-induced endocytosis. Our results identify Sul1 and Sul2 as the first plasma membrane sensors for extracellular sulfate in eukaryotes.

## EXPERIMENTAL PROCEDURES

### 

#### 

##### Yeast Strains and Culture Conditions, Plasmids, and Site-directed Mutagenesis

All *Saccharomyces cerevisiae* strains used in this work have the BY background and are listed in Table 1. Yeast cells were cultured at 30 °C into exponential phase to an *A*_600_ of 1.5–2 in either rich medium (1% (w/v) yeast extract, 2% (w/v) bacterial peptone, 2% glucose) or minimal medium (0.17% (w/v) yeast nitrogen base without amino acids and ammonium sulfate, 0.5% (w/v) ammonium sulfate, auxotrophic supplements as required, and 2% glucose, pH 5.5).

**TABLE 1:** **Yeast strains used in this study**

Yeast strain	Relevant genotype	Source/Reference
BY4742	MATα *his3*Δ *leu2*Δ *lys2*Δ *ura3*Δ	ResGen/Invitrogen
HK 13	BY4742 *sul1*::*KanMX*	This study
BY4742 (*sul2*Δ)	BY4742 *sul2*::*KanMX*	ResGen/Invitrogen
HK 14	MATa *sul1*::*KanMX sul2::KanMX his3*Δ *leu2*Δ *lys2*Δ *ura3*Δ	This study
HK 115	BY4742 *bds1*::*LEU2*	This study
HK 219	BY4742 *sul1*::*KanMX sul2*::*KanMX pep4*::*KanMX*	This study

For sulfur starvation, exponentially grown cells were shifted to sulfur starvation medium (30) containing 4% (w/v) glucose, 15 mm ammonium chloride, 6.6 mm KH_2_PO_4_, 0.5 mm K_2_HPO_4_, 1.7 mm NaCl, 0.7 mm CaCl_2_, 2 mm MgCl_2_, 0.2 mg/liter biotin, 2 mg/liter each of thiamine hydrochloride, pyridoxamine hydrochloride, and calcium pantothenate, 0.5 mg/liter H_3_BO_4_, 0.04 mg/liter CuCl_2_, 0.1 mg/liter KI, 0.05 mg/liter FeCl_3_, 0.2 mg/liter ZnCl_2_, and supplements of amino acids and/or nucleotides, depending on the auxotrophic requirements of the strain. To obtain complete sulfur starvation, the cells were incubated in starvation medium for 2 days under continuous shaking with refreshment of the medium every day. For that purpose, the cells were harvested, washed, and resuspended in fresh sulfur starvation medium. This is done to make sure that the cells are maintained in the glucose-repressed state so that resuspension in new glucose-containing sulfur starvation medium does not stimulate the PKA pathway through the glucose-sensing network that activates cAMP synthesis.

##### Plasmids and Site-directed Mutagenesis

Yeast expression vector pRS316 (*URA3*) (31) and cloning vector pUC18 (32) were used in this work. The C-terminally tagged Sul1 and Sul2 expression constructs were cloned into the pUC18 vector behind their own promoter using the restriction sites XbaI/SalI. These vectors were also used for site-directed mutagenesis, which was performed using Q5 High Fidelity DNA polymerase. The oligonucleotides used are shown in Table 2. Both mutated and wild type Sul1-HA and Sul2-HA expression constructs were cloned into yeast expression vector pRS316 (*URA3*) using the restriction sites KpnI/SalI. All of the plasmids were expressed in a *sul1*Δ *sul2*Δ strain unless otherwise indicated.

**TABLE 2:** **Primers used for site-directed mutagenesis of Sul1,2**

	Mutated residue	Primers
Sul1	D124N	GGGCTATGCTaATTTAGTGGC
		GCCACTAAATtAGCATAGCCC
Sul1	E427Q	TGTCCCTGACCAAcAACTTATTGCGAT
		ATCGCAATAAGTTgTTGGTCAGGGACAA
Sul1	D483N	CTTTATTGTTTAACTaACGCCTTCTTTTTC
		GAAAAAGAAGGCGTtAGTTAAACAATAAAG
Sul1	E538Q	CATCCATTcAAAATGGTATATATTTTG
		CAAAATATATACCATTTTgAATGGATG
Sul2	D305N	CCAAACTGaACGCAGTTTTTG
		CAAAAACTGCGTgCAGTTTGG
Sul2	E443Q	CCCGACCAAcAATTGATTGC
		GCAATCAATTgTTGGTCGGG
Sul2	D519N	CGCTGTATCTaATCTTTTGGCC
		GGCCAAAAGATtAGATACAGCG

##### Origin and Purity of Compounds

d-Glucosamine 2-sulfate was purchased from Carbosynth Ltd. (Compton, UK). Other sulfur-containing compounds were purchased from Sigma-Aldrich. The ^35^S-labeled sodium sulfate and ^3^H-labeled d-glucosamine 2-sulfate were purchased from American Radiolabeled Chemicals. All of the compounds were of ≥98% purity.

##### Biochemical Determinations

For determination of trehalase activity, the cells were first grown up to an *A*_600_ of 1.5 in rich medium or in synthetic medium (supplemented for auxotrophies) and then starved for sulfur in sulfur starvation medium for 2 days. The sulfur-starved cells were harvested after cooling on ice for at least 30 min. The harvested cells were then resuspended in fresh sulfur starvation medium at a concentration of 25 mg of cells/ml. Trehalase activity was measured as described previously (4). Trehalase activity is expressed as nmol of glucose liberated min^−1^ (mg of protein)^−1^. The amount of proteins was measured by the standard Lowry method (33). The amount of glycogen and trehalose was measured as described previously (4). Independent data represent a mean value ± S.D. Representative results are shown for comparisons between collections of interdependent data points (time course measurements). Intracellular cAMP levels were determined as described previously using an Amersham Biosciences [^3^H]cAMP assay kit. The cAMP concentrations are expressed as nmol of cAMP/g wet weight (34).

##### Transport Measurement

Sulfur-starved cells were prepared as described for trehalase activity experiments. Cells were harvested and resuspended at a cell density of 60 mg (wet weight)/ml. Sulfate uptake was measured in 40 μl of cells, preincubated for 10 min at 30 °C, after the addition of 10 μl of 0.5 mm [^35^S]sodium sulfate (specific activity of 2000 cpm/nmol or 0.9 Ci/mol of sodium sulfate) to a final concentration of 0.1 mm, unless stated otherwise. The uptake was stopped after 1 min by adding 5 ml of ice-cold 10 mm sodium sulfate solution, after which the cells were recovered on a glass microfiber filter and washed twice with 5 ml of ice-cold 10 mm sodium sulfate solution. For the blanks, ice-cold sodium sulfate solution was added prior to the addition of [^35^S]sodium sulfate, and the cells were immediately filtered and washed. The filtered cells were counted for ^35^S uptake with a liquid scintillation counter (Beckman Coulter LS6500). Every sample and blank was taken in triplicate, and the average was taken for the final calculation. 10 μl of 0.5 mm [^35^S]sodium sulfate was used to determine the specific activity, and 500 μl of culture was used to determine the dry weight of the cells, which were later used for the final calculation of the sulfate uptake rate. The sulfate uptake rate is expressed as nmol·min^−1^·(mg dry weight)^−1^. For measurement of ^3^H-labeled d-glucosamine 2-sulfate uptake, 40 μl of cell suspension was incubated with 10 μl of 15 mm ^3^H-labeled d-glucosamine 2-sulfate (specific activity of 2000 cpm/nmol or 0.9 Ci/mol of d-glucosamine 2-sulfate; custom synthesized by American Radiolabeled Chemicals).

##### Real-time PCR Analysis

The sulfur-starved cells were harvested at specific time intervals before and after the addition of sulfate. Total RNA was isolated using the phenol-chloroform method and real-time PCR analysis of *SUL1*, *SUL2*, *HSP12*, and *RPL25* expression, and normalization against *ACT1* expression was carried out as described previously (8, 35).

##### Determination of Heat Shock Tolerance

Sulfur-starved cells were harvested and resuspended in fresh sulfur starvation medium containing 4% glucose, and heat shock tolerance was determined as a function of time after the addition of sulfate as described previously (4).

##### Protein Extraction and Western Blot Analysis

For isolation of P13 membrane-enriched fractions, the yeast cells were grown to midexponential phase at 30 °C in appropriate medium and transferred to sulfur starvation medium for 2 days, after which an amount of yeast cells equivalent to 120 *A*_600_ units was harvested at specified time intervals after the addition of sulfate for further processing. P13 membrane-enriched fractions were prepared using first the initial steps of a protocol described previously (36). Instead of further treatment with urea/TCA to precipitate the proteins, the pellets from the first centrifugation step at 13,000 relative centrifugal force (P13 fraction) were resuspended in 400 μl of immunoprecipitation buffer (1% Triton, 150 mm NaCl, 50 mm Na-HEPES, pH 7.5, 5 mm EDTA, 5 mm *N*-ethylmaleimide, and Roche Complete Protease inhibitor tablet), after which immunoprecipitation was performed as described previously (37). The immunoprecipitates were treated with a slurry of 50 μl (1:5) of GE Healthcare (catalog no. 17061801) Protein G-Sepharose beads (pre-equilibrated with immunoprecipitation buffer and anti HA antibody) overnight in a roller drum at 4 °C. The bead-bound immunocomplexes were recovered by centrifugation at 2000 rpm for 1 min. The beads were then washed three times with 1% PBS-T and boiled with 2× sample buffer for 5 min at 95 °C before protein size separation in 4–12% gradient SDS-PAGE (Invitrogen) and subsequent immunoblotting with HRP-coupled 3F10 rat anti HA antibody (Roche Applied Science, catalog no. 12456100).

##### Immunofluorescence Microscopy

For immunodetection of Sul1-HA, Sul2-HA, and their HA-tagged mutant variants, a previously described protocol was used (38). The cell cultures, starved 48 h for sulfur, were fixed for 1 h in 3.7% (v/v) formaldehyde with moderate agitation at 30 °C. The cells were subsequently washed twice with PBS and converted into spheroplasts by incubation for 20 min under mild agitation at 30 °C in PBS with lyticase (Fluka, 92807) (400 units/ml) and 2 μl/ml β-mercaptoethanol. 30 μl of spheroplast suspension was pipetted in separate wells of polylysine-coated slides, dried, and subsequently immersed in −20 °C acetone for 5 min to flatten the cells. The cells were then immediately rehydrated with PBS and blocked with 30 μl of PBS + 3% BSA by incubation in a humid chamber for 30 min. Next, primary antibody was added in PBS with 3% BSA at 1:50 dilution, and the slides were incubated overnight in a humid chamber at 4 °C. The primary antibody (anti-HA rat 3F10; Roche Applied Science) was washed away by five consecutive washes in PBS with 3% BSA, and then secondary antibody (Alexa Fluor 488-coupled goat anti-rat IgG, 2 mg/ml; Molecular Probes, Invitrogen) in a 1:500 dilution was added in the same medium. Slides were incubated with secondary antibody for 4 h at room temperature, after which the secondary antibody was washed off by 5 volumes of PBS. PBS was removed by aspiration, and 5 μl of anti-fading Fluoromont medium (Sigma) was added to each well. The slides were sealed with a coverslip and visualized by confocal microscopy. Imaging was carried out using an Olympus FV1000 confocal laser scanning biological microscope, and images were processed with the accompanying software, FV10-ASW version 2.0.

##### Reproducibility of the Results

All experiments were repeated independently three to four times and always included at least two technical replicates. S.D. values for biological replicates are shown as *error bars* for comparisons between independent data points (determination of transport rate). Representative results are shown for comparisons between collections of interdependent data points (time course measurements). The rate and maximal extent of sulfate-induced responses were variable between different experiments, but the differences reported between controls and samples were consistently reproducible.

## RESULTS

### 

#### 

##### Sul1 and Sul2 Are Required for Activation of PKA Targets upon Addition of Sulfate to Sulfur-starved Cells

**FIGURE 1.**
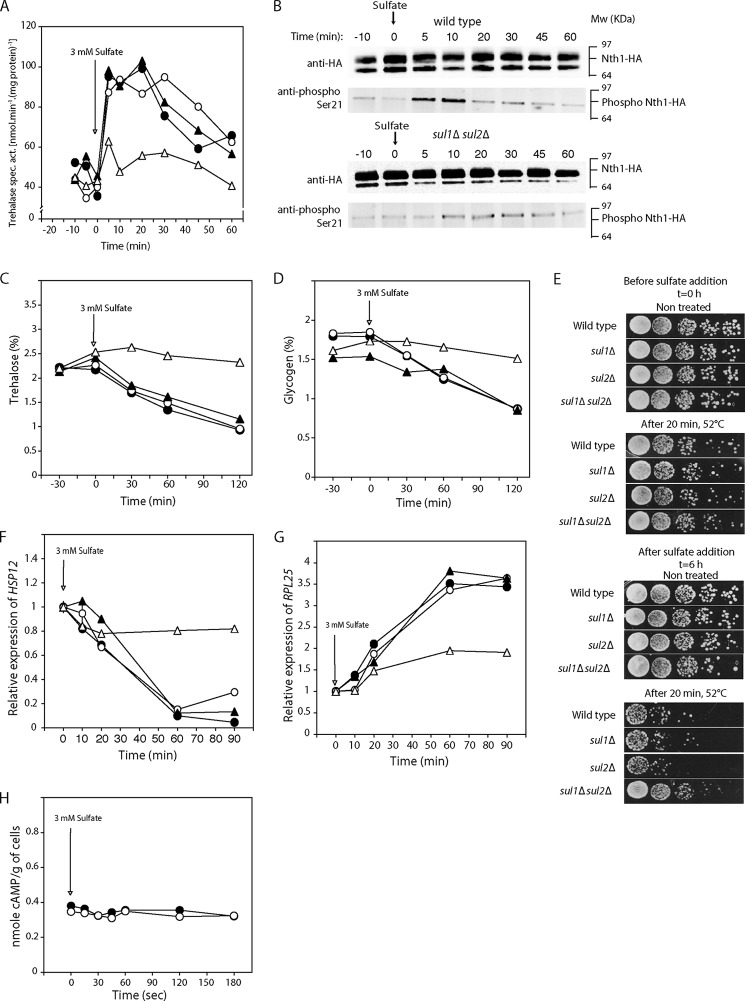
**The addition of sulfate to sulfate-starved cells activates PKA targets through Sul1 and Sul2.** *A*, activation of trehalase. *B*, phosphorylation of Nth1-HA detected by Western blot with phospho-specific antibodies in wild type cells and mutant *sul1*Δ *sul2*Δ cells. The phospho-specific antibody raised against phosphorylated Ser-21 of Nth1 was used to monitor the phosphorylation status of Nth1-HA. Anti-HA antibody was used to monitor the level of Nth1-HA. *C*, mobilization of trehalose. *D*, mobilization of glycogen. *E*, loss of heat stress tolerance. Cells were subjected to a heat shock at 52 °C for 20 min before or 6 h after the addition of sulfate, and serial dilutions of the cultures were then spotted on plates with rich medium and allowed to grow at 30 °C. Untreated cells were analyzed in parallel as a control. Shown are expression levels relative to the expression at time 0 of heat shock protein gene *HSP12* (*F*) and ribosomal protein gene *RPL25* (*G*), upon the addition of 3 mm sulfate to sulfur-starved cells. Strains are shown as follows: wild type (●), *sul1*Δ (○), *sul2*Δ (▴), and *sul1*Δ *sul2*Δ (▵). *H*, intracellular level of cAMP in wild type (●) and *sul1*Δ *sul2*Δ (○) cells at the indicated times before and after the addition of 3 mm sulfate to sulfur-starved cells.

Previous work has shown that sulfate addition to sulfur-starved cells on a glucose-containing medium triggers activation of trehalase, a classical read-out for rapid PKA activation *in vivo* in yeast (27). We now show that this sulfate-induced activation of PKA requires one of the two sulfate transporters, either Sul1 or Sul2 (Fig. 1*A*). Using site- and phospho-specific antibodies, we show that activation of trehalase is correlated with phosphorylation on the PKA consensus site, Ser-21 (Fig. 1*B*), similar to trehalase activation by glucose and nitrogen sources in appropriately starved cells (28). We next confirmed the requirement of Sul1 or Sul2 for sulfate-induced activation of PKA using several other *in vivo* read-outs for activation of the PKA pathway. After the addition of sulfate to sulfur-starved cells, the carbohydrates trehalose (Fig. 1*C*) and glycogen (Fig. 1*D*) were mobilized, heat stress tolerance dropped (Fig. 1*E*), expression of the heat shock gene *HSP12* was down-regulated (Fig. 1*F*), and expression of the ribosomal gene *RPL25* was up-regulated (Fig. 1*G*). All of these sulfate-induced processes required the presence of either Sul1 or Sul2 (Fig. 1, *A–G*). Consistent with previous results (27), sulfate activation of the PKA targets in sulfur-starved cells with BY genetic background was not associated with an increase in the cAMP level (Fig. 1*H*). In the *sul1*Δ *sul2*Δ strain, there was a residual phosphorylation signal for trehalase (Fig. 1*B*) that correlated with a residual increase in its activity (Fig. 1*A*). Also for the other PKA targets, trehalose mobilization (Fig. 1*C*), glycogen mobilization (Fig. 1*D*), loss of heat stress tolerance (Fig. 1*E*), reduced expression of *HSP12* (Fig. 1*F*), and enhanced expression of *RPL25* (Fig. 1*G*), there was a residual effect in the *sul1*Δ *sul2*Δ strain. This residual effect is probably due to uptake of sulfate through a third sulfate carrier with much lower affinity for sulfate than Sul1 and Sul2 because the *sul1*Δ *sul2*Δ strain can grow with a very high sulfate concentration (>20 mm) as the sole source of sulfur in the medium.^2^

##### Screening of a Collection of Sulfur-containing Compounds for Non-transported Signaling Agonists

Although the previous results clearly establish a requirement of either Sul1 or Sul2 for sulfate-induced activation of PKA targets, they do not allow us to distinguish between a mere requirement for sulfate entering the cells or for the functioning of Sul1 and Sul2 as sulfate receptors, triggering activation of the PKA pathway. Hence, we decided to perform a screen for non-transported sulfur-containing compounds able to activate the PKA pathway in a Sul1- or Sul2-dependent way, similar to previous work in which l-Leu-Gly (5) and glycerol 3-phosphate (7) were identified as non-transported signaling agonists of the Gap1 and Pho84 transceptors, respectively. For that purpose, we tested trehalase activation in the wild type and *sul1*Δ *sul2*Δ strains upon the addition of various sulfur-containing compounds and sulfate analogues to sulfur-starved cells (Table 3).

**TABLE 3:** **Overview of trehalase activation, support of growth as sole source of sulfur, and uptake for different sulfur-containing compounds and sulfate analogues**

Compounds	Trehalase activation		Support of growth		Uptake	
	Wild type cells	*sul1*Δ *sul2*Δ	Wild type cells	*sul1*Δ *sul2*Δ	Wild type cells	*sul1*Δ *sul2*Δ
2-Aminoethyl hydrogen sulfate	+	−	+	−	Not tested	Not tested
d-Glucosamine 2 sulfate	+	−	−	−	−	−
Glutathione	+	+	+	+	Not tested	Not tested
Methionine	+	+	+	+	Not tested	Not tested
Methionine sulfoxide	+	+	+	+	Not tested	Not tested
Methyl methionine sulfonium	+	+	+	+	Not tested	Not tested
Methyl sulfate sodium salt	+	−	+	−	Not tested	Not tested
*S*-methyl methionine	+	+	+	+	Not tested	Not tested
Sodium bisulfite	−	−	−	−	Not tested	Not tested
Sodium molybdate	+	−	−	−	Not tested	Not tested
Sodium selenate	+	−	−	−	Not tested	Not tested
Sodium sulfate	+	−	+	−	+	−
				(at concentration < 3 mm)		(at concentration < 3 mm)
				+		+
				(at concentration > 3 mm)		(at concentration > 3 mm)
Sodium sulfite	+	+	+	+	Not tested	Not tested
Sodium thiosulfate	+	+	+	+	Not tested	Not tested

**FIGURE 2.**
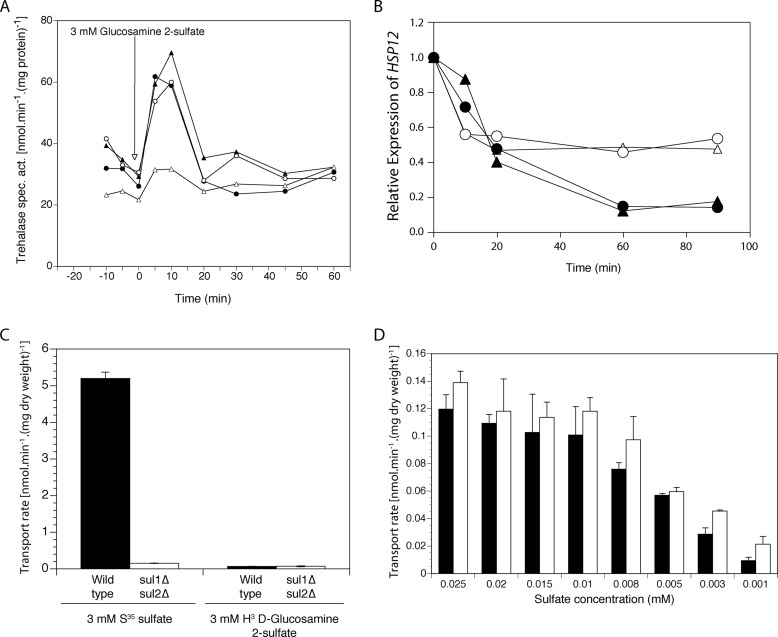
**d-Glucosamine 2-sulfate activates the PKA target trehalase, whereas its uptake is below the detection level.** *A*, activation of trehalase in sulfur-starved wild type (●), *sul1*Δ (○), *sul2*Δ (▴), and *sul1*Δ *sul2*Δ (▵) cells after the addition of 3 mm d-glucosamine 2-sulfate. *B*, relative expression of *HSP12* in sulfur-starved *sul1*Δ *sul2*Δ cells expressing Sul1-HA (*circles*) and Sul2-HA (*triangles*) upon the addition of 3 mm sulfate (*closed symbols*) or 3 mm d-glucosamine 2-sulfate (*open symbols*). *C*, uptake rate of ^3^H-labeled d-glucosamine 2-sulfate and ^35^S-labeled sodium sulfate in wild type (*black bars*) and *sul1*Δ *sul2*Δ (*white bars*). *D*, uptake rate of different concentrations of ^35^S-labeled sodium sulfate in the presence (*black bars*) and absence (*white bars*) of 10 mm d-glucosamine 2-sulfate (*G2S*). *Error bars*, S.D.

We found three compounds able to activate trehalase in a Sul1- or Sul2-dependent manner: methyl sulfate, 2-aminoethyl hydrogen sulfate (Table 3), and d-glucosamine 2-sulfate (Fig. 2*A*). To confirm the Sul1/Sul2-dependent signaling nature of d-glucosamine 2-sulfate, we measured the gene expression level of *HSP12* and show that it drops upon the addition of 3 mm sulfate or 3 mm d-glucosamine 2-sulfate in cells only expressing Sul1-HA or Sul2-HA (Fig. 2*B*).

To investigate whether d-glucosamine 2-sulfate was taken up into the yeast cells, we made use of custom-ordered ^3^H-labeled d-glucosamine 2-sulfate and found that this compound lacks any detectable uptake by wild type or *sul1*Δ *sul2*Δ cells (Fig. 2*C*). Hence, the apparent absence of uptake prevents any possible use as a sole source of sulfur. Furthermore, concentrations as high as 5, 10, 25, and 50 mm d-glucosamine 2-sulfate did not cause inhibition of uptake by Sul1 or Sul2 of 0.005 mm sulfate (results not shown). Only with sulfate concentrations below the *K_m_* of sulfate transport (4.5 μm for Sul1 and 10 μm for Sul2) (21), we could detect significant inhibition with 10 mm d-glucosamine 2-sulfate (Fig. 2*D*). This appears to indicate that d-glucosamine 2-sulfate does not interact with the canonical sulfate-binding site of Sul1 or Sul2 or that it has an extremely low affinity for it (>50 mm). From these results, we can conclude that d-glucosamine 2-sulfate is a non-transported signaling agonist of Sul1 and Sul2 and that the latter therefore function as both transporters and receptors, or transceptors. Sul1 and Sul2 are the first plasma membrane sensors for extracellular sulfate identified to date.

The activation of trehalase by d-glucosamine 2-sulfate (Fig. 2*A*) was more transient than with sulfate (Fig. 1*A*). Similarly, reduced expression of *HSP12* was shorter lived with d-glucosamine 2-sulfate than with sulfate (Fig. 2*B*). This may indicate that the initial burst in activation of the PKA pathway is caused solely by transceptor signaling, whereas maintenance of high activity over a longer time is dependent on sulfate metabolism.

**FIGURE 3.**
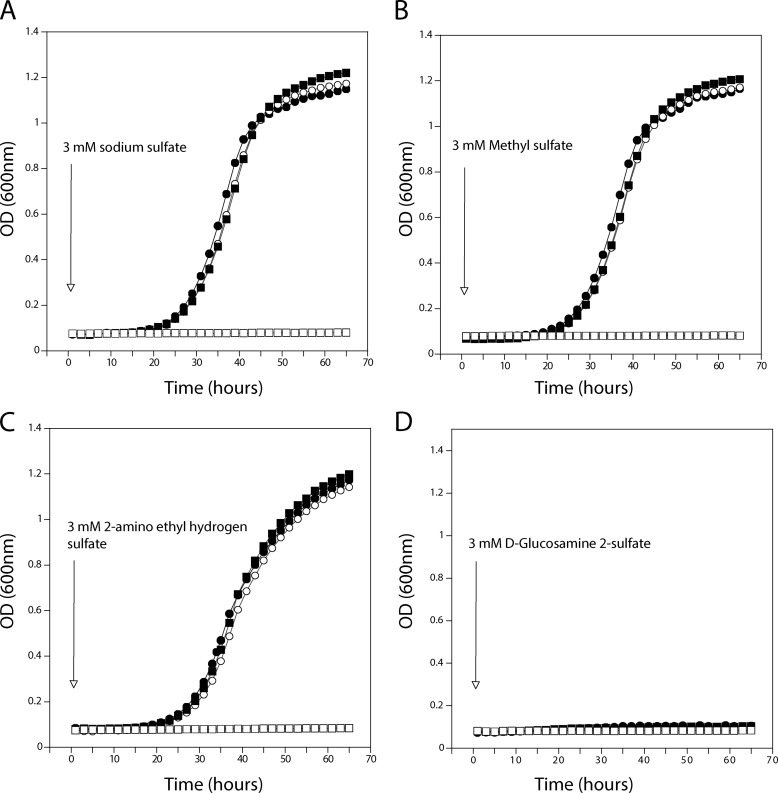
**Cell growth with different sulfate-containing compounds as sole source of sulfur.** Wild type (●), *sul1*Δ (○), *sul2*Δ (■), and *sul1*Δ *sul2*Δ (□) cells were starved for sulfur and then transferred to medium containing a 3 mm concentration of the indicated sulfate-containing compounds (*A–D*).

**FIGURE 4.**
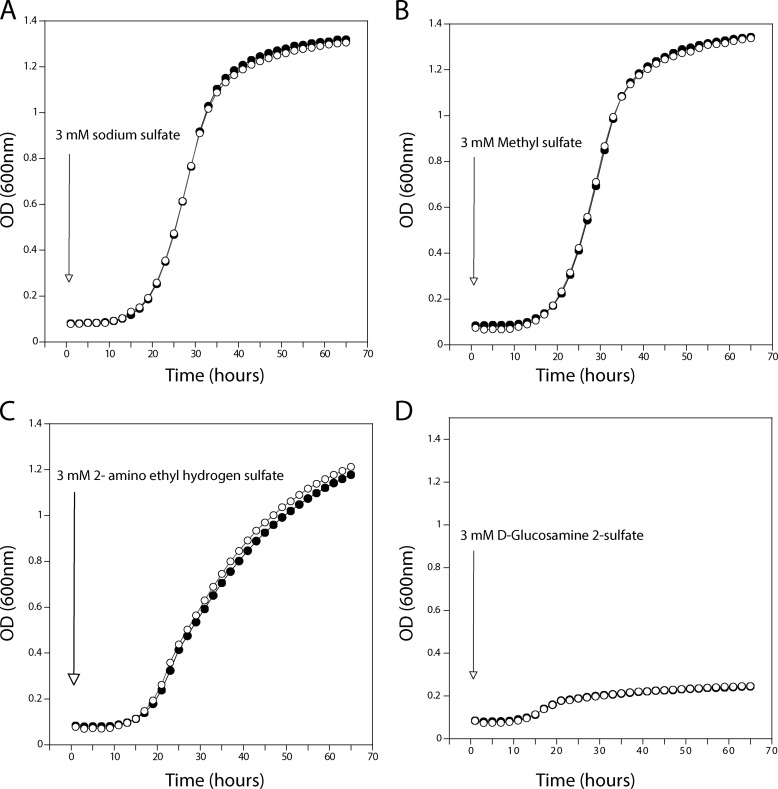
**The *BDS1* gene does not influence growth on various sulfate-containing compounds.** Wild type (●) and *bds1*Δ (○) cells were starved for sulfur and then transferred to medium containing a 3 mm concentration of the indicated sulfate-containing compounds (*A–D*).

We also investigated whether the sulfate analogues could be used as a sole source of sulfur by yeast cells. We found that wild type (and also *sul1*Δ and *sul2*Δ single deletion) strains, but not the *sul1*Δ *sul2*Δ strain, were able to grow using methyl sulfate and 2-aminoethyl hydrogen sulfate as the sole source of sulfur. This indicated that methyl sulfate and 2-aminoethyl hydrogen sulfate were apparently transported into the cells by Sul1 and Sul2 and also further metabolized to support growth. On the other hand, d-glucosamine 2-sulfate did not support growth of any of the strains tested (Table 3 and Fig. 3). This is consistent with the observation that d-glucosamine 2-sulfate was not taken up by yeast cells. In order to rule out that growth in methyl sulfate and 2-aminoethyl hydrogen sulfate was due to release of sulfate from these compounds in the medium by secreted sulfatases, we deleted the only known sulfatase gene, *BDS1*, present in *S. cerevisiae* (39). The *bds1*Δ strain showed similar growth in methyl sulfate and 2-aminoethyl hydrogen sulfate as sole sources of sulfur as the wild type strain (Fig. 4), indicating that growth was not due to extracellular release of sulfate from these compounds but rather to uptake and intracellular assimilation.

##### Mutagenesis of Putative H^+^-binding Amino Acid Residues in Sul1 and Sul2 Identifies a Residue Required for Transport but Not for Signaling

Sul1 and Sul2 are H^+^-symporters, which were reported to use three H^+^ per sulfate ion transported (40). We next investigated whether we could abolish sulfate transport without affecting sulfate signaling by mutagenizing putative H^+^-binding residues. The rare charged amino acid residues within transmembrane domains of membrane symporters are thought to bind the co-transported ions during their passage through the transporter. Because the symport mechanism is essential for transport, such residues are usually conserved in the transporter family (41–44).

**FIGURE 5.**
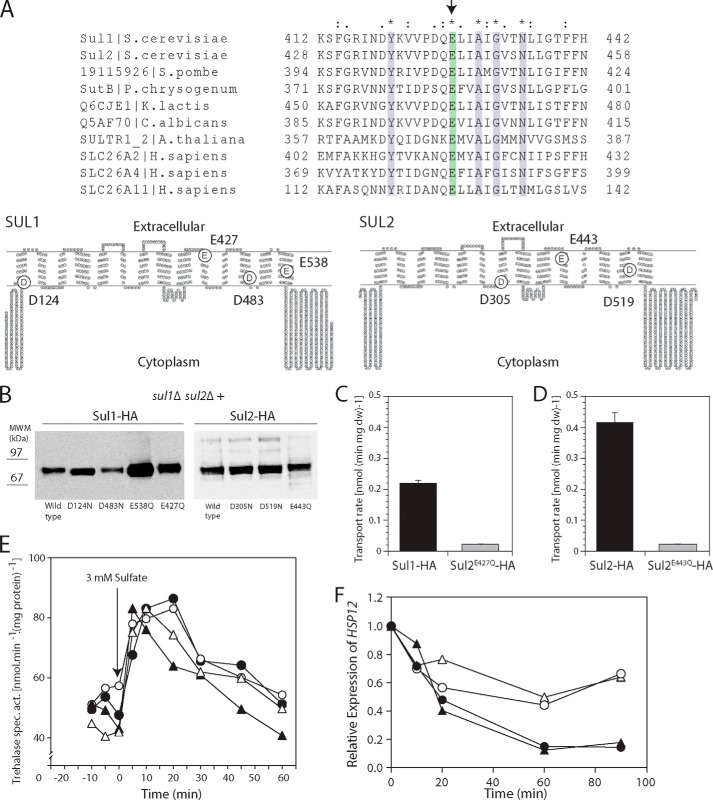
**Mutagenesis of the putative proton binding sites, Glu-427 in Sul1 and Glu-443 in Sul2, blocks the uptake of sulfate but does not affect the signaling function.** *A*, ClustalX sequence alignment of sulfate transporters from different organisms showing the conserved glutamic acid residue (Glu-427 in Sul1 and Glu-443 in Sul2) (in *green*) and other conserved residues (in *lavender*). *Bottom*, the position of all mutagenized residues, including Glu-427 in Sul1 and Glu-443 in Sul2, is indicated on the predicted topology of Sul1 and Sul2. *B*, Sul1,2-HA expression level as detected by Western blot of immunoprecipitated Sul1-HA, Sul2-HA, and their mutant forms in membrane-enriched P13 fractions isolated from cell cultures before and after the addition of 3 mm sulfate. *C*, uptake rate of 0.1 mm [^35^S]sulfate in Sul1-HA-expressing (*black bar*) and Sul1^E427Q^-HA-expressing (*gray bar*) sulfur-starved cells. *D*, uptake rate of 0.1 mm [^35^S]sulfate in Sul2-HA-expressing (*black bar*) and Sul2^E443Q^-HA-expressing (*gray bar*) sulfur-starved cells. *E*, activation of trehalase in Sul1-HA (●), Sul1^E427Q^-HA (○), Sul2-HA (▴), and Sul2^E443Q^-HA (▵) upon the addition of 3 mm sulfate. *F*, relative expression of *HSP12* in sulfur-starved *sul1*Δ *sul2*Δ cells expressing Sul1-HA (●), Sul1^E427Q^-HA (○), Sul2-HA (▴), and Sul2^E443Q^-HA (▵) upon the addition of 3 mm sulfate. *Error bars*, S.D.

The hidden Markov model-based topology prediction algorithm, PHOBIUS (45), predicted 12 and 11 transmembrane domains for Sul1 and Sul2, respectively. However, homology modeling (threading) using the HHpred algorithm showed that Sul1,2 are structurally most similar to the *Escherichia coli* uracil UraA transporter, the crystal structure of which showed 14 TMDs^3^ (46) (also see “Discussion”). Based on topology predictions and the similarity with UraA, we screened the 14 putative transmembrane domains of the Sul1,2 transporters for charged residues, in particular Glu and Asp residues, which are the best candidates for H^+^ binding and symport. We found six residues in Sul1 and five residues in Sul2, located within or very close to predicted transmembrane domains (Table 4). Alignment of sulfate transporters from different organisms revealed that Glu-406 and Glu-427 in Sul1 and Glu-422 and Glu-443 in Sul2, located in TMD8 and TMD9, respectively, were absolutely conserved in other sulfate transporters from different organisms (Fig. 5*A*) (47). Furthermore, residues Glu-406 and Glu-422 in Sul1 and Sul2, respectively, correspond to the His-245 residue proposed to interact with H^+^ in the UraA structure (46). We therefore selected to mutate these residues from Sul1 and two from Sul2 for site-directed mutagenesis (Table 4).

**TABLE 4:** **Putative proton binding sites in Sul1 and Sul2** Corresponding amino acid residues in Sul1 and Sul2 are indicated in the same row.

Sul1		Sul2		TMD
Predicted site	Mutant allele analyzed	Predicted site	Mutant allele analyzed	
Asp-124	D124N	Asp-140		1
		Asp-305	D305N	7
Glu-406		Glu-422		9
Glu-427	E427Q	Glu-443	E443Q	10
Asp-483	D483N			12
Asp-503		Asp-519	D519N	13
Glu-538	E538Q			14

**FIGURE 6.**
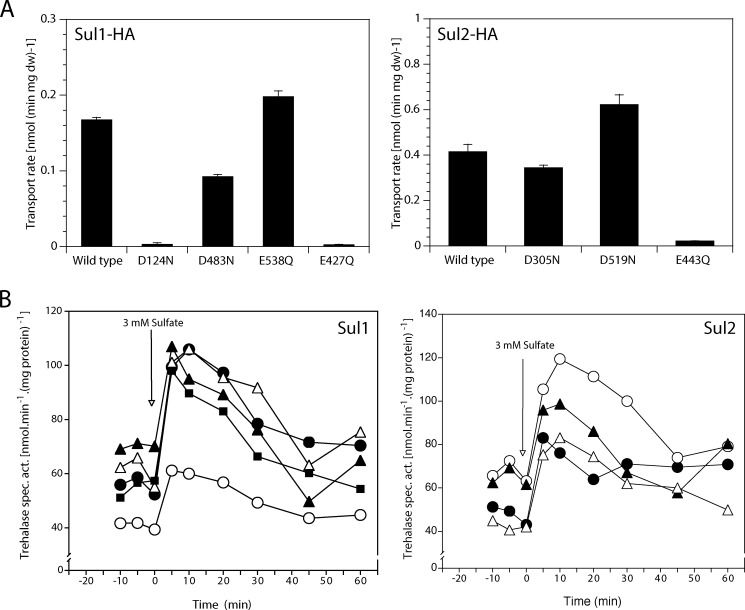
**Sulfate transport and sulfate-induced PKA signaling in cells expressing Sul1 and Sul2 versions mutated in different putative proton-binding residues.** *A*, uptake rate of 0.1 mm [^35^S]sulfate in sulfur-starved cells expressing wild type Sul1-HA, Sul2-HA, or a mutant form. *B*, trehalase activation upon the addition of 3 mm sulfate to sulfur-starved cells expressing the following: Sul1-HA (●), Sul1^D124N^-HA (○), Sul1^D483N^-HA (▴), Sul1^E538Q^-HA (▵), and Sul1^E427Q^-HA (■) (*left*) and Sul2-HA (●), Sul2^D305N^-HA (○), Sul2^D519N^-HA (▴), and Sul2^E443Q^-HA (▵) (*right*). *Error bars*, S.D.

Glu and Asp residues were individually mutagenized to uncharged Gln and Asn residues, respectively. Plasmids encoding C-terminally HA-tagged versions of Sul1 and Sul2 containing the respective mutation (Sul1^mut^-HA or Sul2^mut^-HA) were constructed, transformed in the *sul1*Δ *sul2*Δ strain, and tested for proper expression of the mutant proteins by Western blot analysis of membrane-enriched (P13) protein extracts (Fig. 5*B*). Strains expressing these proteins were then investigated for their sulfate transport and signaling activities in sulfur-starved cells and compared with a strain expressing the wild type construct. The sulfate uptake rate with different mutant forms of Sul1 and Sul2 was significantly reduced (Figs. 5 (*C* and *D*) and 6*A*). Sul1^E427Q^-HA, Sul1^D124N^-HA, and Sul2^E443Q^-HA showed negligible uptake activity (Figs. 5 (*C* and *D*) and 6*A*) despite being properly expressed as assessed by Western blot analysis of the HA-tagged versions present in membrane-enriched fractions of sulfur-starved cells (Fig. 5*B*).

We next investigated sulfate-induced signaling to the PKA pathway in sulfur-starved cells of strains expressing the mutant Sul1 or Sul2 proteins. This showed that the non-transporting mutant forms Sul1^E427Q^-HA and Sul2^E443Q^-HA had completely retained their capacity to mediate activation of the PKA target trehalase (Figs. 5*E* and 6*B*). In contrast, the only other transport-deficient mutant identified, Sul1^D124N^-HA, failed to activate trehalase to the wild type level (Fig. 6*B*). We confirmed the ability of Sul1^E427Q^-HA and Sul2^E443Q^-HA to activate the PKA pathway by measuring the reduced expression of *HSP12* upon the addition of sulfate (Fig. 5*F*). The reduced expression of *HSP12* took place in a similar way as observed previously for the addition of d-glucosamine 2-sulfate to sulfur-starved cells (Fig. 2*B*). The rapid initial effect was present, but the long term effect was reduced. As previously mentioned, reduction of the long term effect may be due to the absence of sulfate assimilation. Hence, both Sul1^E427Q^-HA and Sul2^E443Q^-HA had retained signaling activity despite the loss of transport activity and can thus function as pure sulfate sensors. This further underscored that both Sul1 and Sul2 are true transceptors, in which the transport function can be uncoupled from the signaling function.

##### Sulfate-mediated Transcriptional and Post-translational Down-regulation of Sul1 and Sul2

**FIGURE 7.**
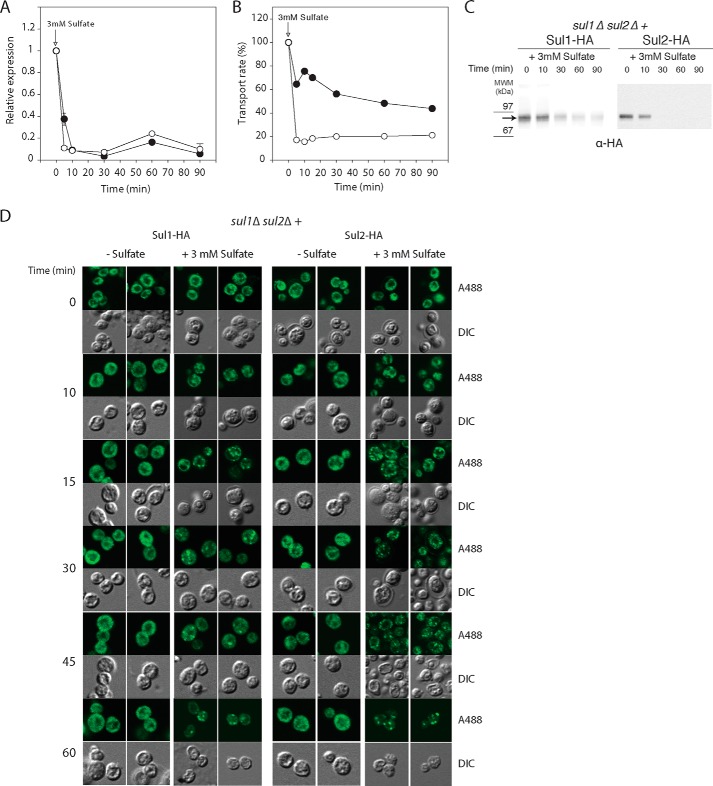
**Sulfate transceptors Sul1 and Sul2 are regulated at the transcriptional and post-transcriptional level.** *A*, relative mRNA levels of *SUL1* (●) and *SUL2* (○) before and after the addition of 3 mm sulfate to sulfur-starved cells. *B*, uptake rate of [^35^S]sulfate in Sul1-HA-expressing (●) and Sul2-HA-expressing (○) cells before and after the addition of sulfate to sulfur-starved cells. The cells were harvested at the indicated time points, and the short term uptake rate was then measured with a 1-min uptake assay using radioactive sulfate. *C*, immunoprecipitation of Sul1-HA and Sul2-HA from membrane-enriched (P13) fractions, isolated before and at different time intervals after the addition of 3 mm sulfate. The immunoprecipitated samples were blotted and immunodetected with anti-HA antibody. The predicted size of the Sul1-HA and Sul2-HA proteins is indicated with an *arrow. D*, localization of Sul1-HA or Sul2-HA was monitored in the absence of sulfate (−) and after the addition of 3 mm sulfate (+) by immunofluorescence and confocal microscopy imaging. HA-tagged Sul1 and Sul2 proteins were detected by treatment with anti-HA rat primary antibody followed by treatment with Alexa Fluor 488-conjugated anti-rat secondary antibody. *DIC*, differential interference contrast.

Starvation of yeast cells for sulfur causes strong induction of *SUL1* and *SUL2* and higher levels of the Sul1 and Sul2 high affinity sulfate transporters at the plasma membrane, whereas the addition of sulfate causes rapid down-regulation of sulfate uptake (20, 25). We confirmed that a strong drop in the mRNA level for both *SUL1* and *SUL2* takes place shortly after the addition of 3 mm sulfate to cells starved for sulfur, indicating a tight negative regulation of the sulfate transporters at the transcriptional level and/or sudden stimulation of mRNA breakdown upon substrate availability (Fig. 7*A*). Moreover, upon the readdition of sulfate to sulfur-starved cells, the sulfate uptake rate also decreased rapidly and dramatically over time. In particular, the uptake rate of a *sul1*Δ *sul2*Δ strain expressing only Sul1-HA dropped to 60% in only 5 min and then slowly but progressively declined further, suggesting the involvement of two different inactivation processes. The uptake rate of the *sul1*Δ *sul2*Δ strain expressing only Sul2-HA dropped much more sharply. It declined to below 20% within 5 min and then remained constant at this low level (Fig. 7*B*). These observations indicate a tight regulation of sulfate transporting capacity, possibly both by allosteric regulation and intracellular sorting, as well as a differential regulation of Sul1 and Sul2 upon the readdition of the substrate.

Because the sharpest decrease in sulfate transport by Sul1 or Sul2 is observed within 5 min after the readdition of sulfate, it is unlikely that this decrease is solely due to transcriptional repression or stimulation of mRNA breakdown; rather, it suggests a post-translational mode of regulation, such as initiation of substrate-induced endocytosis. To verify this, we next studied the stability of Sul1 and Sul2 at the plasma membrane upon the addition of 3 mm sulfate to sulfur-starved cells in two ways (Fig. 7, *C* and *D*). Because Sul1 and Sul2 N- or C-terminal GFP fusion proteins were constitutively sorted to the vacuole, we used Western blot analysis to detect Sul1-HA or Sul2-HA in immunoprecipitates from membrane-enriched protein (P13) fractions of *sul1*Δ *sul2*Δ cells expressing each version. A progressive disappearance of each of the transceptors from the membrane fractions was observed in this way, which was detectable already 10 min after the addition of sulfate (Fig. 7*C*). This suggests that both Sul1 and Sul2 are endocytosed. The faster disappearance of Sul2-HA, in comparison with Sul1-HA, is in agreement with the faster decrease in transport activity also observed for Sul2-HA (Fig. 7*B*). The rate of disappearance of the Sul-HA signals was somewhat variable, but the relative difference was consistent. For instance, Sul2-HA always disappeared faster than Sul1-HA. Detection of Sul1-HA and Sul2-HA by immunofluorescence microscopy confirmed that both transceptors are initially localized at the cell surface and that already within 10 min after the addition of 3 mm sulfate, they are progressively internalized, resulting in their transient accumulation in cytosolic punctate structures, consistent with endosomes (Fig. 7*D*). Taken together, our data indicate that the Sul1 and Sul2 transceptors are present in a high concentration at the plasma membrane in sulfur-starved cells and are subjected to sulfate-triggered reduction of transcript levels as well as post-translational down-regulation by endocytosis.

##### Uncoupling of Signaling from Endocytosis in the Sulfate Transceptors

**FIGURE 8.**
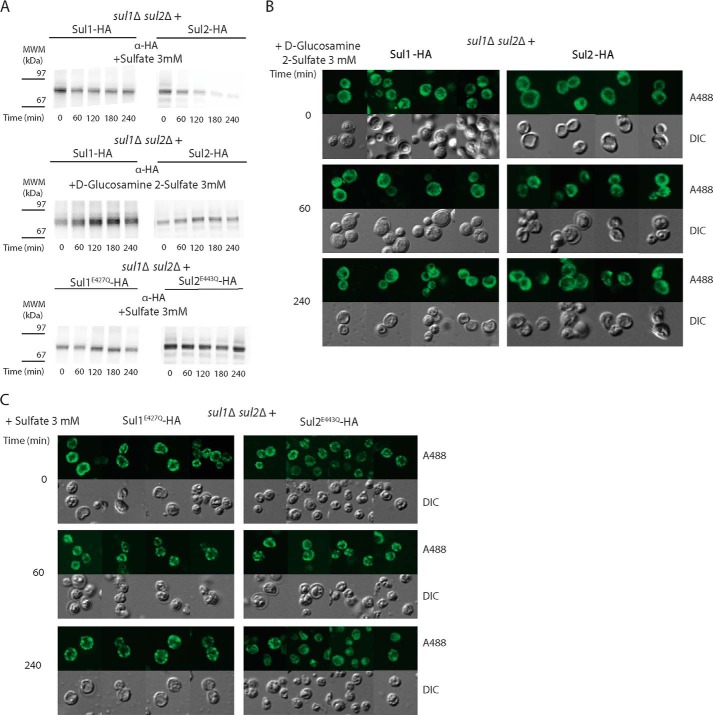
**Uncoupling signaling from endocytosis in sulfate transceptors.** *A*, Western blot detection of immunoprecipitated HA-tagged Sul proteins. Shown are Sul1-HA or Sul2-HA in membrane-enriched P13 fractions isolated from cells before and after the addition of 3 mm sulfate or 3 mm d-glucosamine 2-sulfate. Shown are Sul1^E427Q^-HA and Sul2^E443Q^-HA before and after the addition of 3 mm sulfate. *B* and *C*, localization of HA-tagged Sul proteins as detected by immunofluorescence and confocal microscopy imaging. *B*, Sul1-HA or Sul2-HA before and after the addition of 3 mm d-glucosamine 2-sulfate. *C*, Sul1^E427Q^-HA or Sul2^E443Q^-HA before and after the addition of 3 mm sulfate. In all immunofluorescence experiments, HA-tagged Sul proteins were detected with anti-HA rat primary antibody and Alexa Fluor 488-conjugated anti-rat secondary antibody. *DIC*, differential interference contrast.

We next investigated whether signaling and transport could be uncoupled from endocytosis using the non-transported signaling agonist, d-glucosamine 2-sulfate. The addition of d-glucosamine 2-sulfate did not cause any disappearance of Sul1-HA or Sul2-HA in immunoprecipitates of membrane enriched P13 fractions (Fig. 8*A*). This was confirmed with immunofluorescence microscopy, which showed no detectable change in the intracellular localization of Sul1-HA and Sul2-HA up to 240 min after the addition of d-glucosamine 2-sulfate (Fig. 8*B*). These results show that signaling is either not a trigger or at least not sufficient to trigger endocytosis.

Next, we tested whether the two transport-defective but signaling variants, Sul1^E427Q^-HA and Sul2^E443Q^-HA, still displayed sulfate-induced endocytosis. Arguing against endocytosis is that their presence in P13 fractions from *sul1*Δ *sul2*Δ cells expressing either mutant was unchanged upon sulfate addition (Fig. 8*A*). The absence of endocytosis was also confirmed by immunofluorescence microscopy. The localization of these mutant proteins was more irregular and dotted but always near the periphery of cells and did not change even 240 min after the addition of sulfate (Fig. 8*C*). For this reason, and also because of maintenance of their signaling activity, we believe that these mutant forms are located at the plasma membrane and are insensitive to sulfate-induced endocytosis. Their particular distribution pattern may be related to association with specific areas in the plasma membrane, which is not commonly seen for wild type Sul1 and Sul2. Hence, mutagenesis of the putative H^+^-binding residues does not abolish signaling activity, although it apparently causes additional effects on the structure of the transceptors, which prevents not only sulfate transport but also their sulfate-induced internalization.

## DISCUSSION

### 

#### 

##### Is Exit from Nutrient Starvation Arrest Always Associated with Transceptor Activation?

The high affinity transporters that are expressed by yeast and other microorganisms upon limitation or deprivation of their nutrient substrate have generally been considered to function as scavenger transporters, taking up minute amounts of substrate from the medium. When ample substrate again becomes available to the cells and they are thus relieved from their growth limitation or arrest, such transporters are usually rapidly down-regulated both at the transcriptional and post-translational level. The latter often involves endocytic internalization and sorting to the vacuole for breakdown (48–53). Our previous work has already revealed that certain high affinity transporters, induced upon limitation or starvation for their own substrate, play an active role as nutrient sensors for activation of PKA during exit from nutrient deprivation-induced arrest of cell proliferation. Because of their dual transporter/receptor function, these plasma membrane proteins have been called “transceptors” (2). In *S. cerevisiae*, Gap1 (4, 5), Mep2 (8), and Pho84 (6, 7) have been shown to play a role in rapid activation of the PKA pathway during exit from growth arrest caused by nitrogen (Gap1 and Mep2) or phosphate (Pho84) deprivation in fermenting cells. This also makes physiological sense because PKA is known to stimulate fermentation and growth. It has remained unclear, however, how general the concept of transceptor activation of the PKA pathway is. Starvation for any essential nutrient is well known to cause growth arrest in the G_1_ phase of the cell cycle and entrance into the G_0_ stationary phase. This raises the question of whether exit from nutrient starvation-induced cell cycle arrest is always associated with signaling by one or more transceptors. In the present paper, we show that sulfate-induced exit from stationary phase is also associated with signaling by the Sul1 and Sul2 transceptors. The situation for sulfate is somewhat different from that of the previously described transceptors because Sul1 and Sul2 are the only sulfate transporters known in yeast. This indicates that the main transporters for a given nutrient may all function as transceptors. The demonstration of transceptor signaling with sulfate seems to support the concept that nutrient transceptor signaling during exit from starvation arrest is a general phenomenon occurring with all (essential) nutrients.

##### The Yeast Sul1 and Sul2 Sulfate Transporters Are the First Sulfate Transceptors

The main challenge in demonstrating that a nutrient transporter acts as a transceptor is to distinguish between the transporter itself and the internalized nutrient triggering the signaling. It is not easy to find mutations in a transceptor that abolish only one of the two functions. Usually, mutant alleles display both reduced transport and reduced signaling, preventing any clear conclusion to be drawn. In this paper, we have made a conceptual advance in this respect by exploring systematically amino acid residues that are good candidates for binding a symported cation. This resulted in identification of a specific allele both for Sul1 and Sul2 unable to transport but maintaining the capacity for signaling. Hence, we may have discovered a rational approach for separating transport and signaling in a transceptor by site-directed mutagenesis. It is also not easy to identify substrate analogs that are transported without triggering signaling or triggering signaling without being transported. Of all compounds tested, only d-glucosamine 2-sulfate was able to fulfill one of the two; it triggered signaling without being transported. Unexpectedly, high concentrations of d-glucosamine 2-sulfate only caused significant inhibition of sulfate uptake below its *K_m_*. This indicates that the main interaction site of d-glucosamine 2-sulfate with Sul1 or Sul2 is probably not through the canonical sulfate-binding site. The simplest explanation would be that d-glucosamine 2-sulfate binds with low affinity to an unknown site, possibly at an outward facing domain and thus induces the conformational change required for signaling, in a way distinct from sulfate. This apparent paradox may reflect that the conformation of transporters is much more flexible than suggested by the outward open/occluded/inward open models. This is consistent with recent findings where specific transporters have been shown to possess distinct binding sites and translocation trajectories for some of their substrates or ligands (54, 55). In particular, the UapA xanthine-uric acid transporter of *Aspergillus nidulans* has been shown to be able to bind with high affinity and transport the purine analogue allopurinol, but allopurinol binding neither inhibits binding and transport of the physiological substrates nor induces UapA transport-dependent endocytosis, in contrast to uric acid or xanthine (54). Although allopurinol is transported and d-glucosamine 2-sulfate is not, both compounds provide an example where competitive inhibition is not observed under conditions where it was expected.

In the second approach, we mutagenized putative H^+^-binding residues in transmembrane domains with the aim of abolishing transport and maintaining signaling in a mutant Sul1,2 transceptor. We identified Glu-427 in Sul1 and the corresponding Glu-443 in Sul2 as being required for transport but not for signaling. Another residue, Asp-124 in Sul1, also conserved in Sul2 at the position Asp-140, was necessary for both transport and signaling. The strong conservation of these residues is consistent with a crucial role in the transport mechanism (47). The non-transporting signaling forms of the transceptors Sul1 and Sul2 can be compared with the well established non-transporting amino acid sensor, Ssy1 (56–58), and glucose sensors, Snf3 and Rgt2 (59). These proteins show strong sequence similarity with transporters but have lost the capacity to transport and function instead as pure sensors. Interestingly, a recent report shows that mutations in the residue Glu-357 in another member of the sulfate transporter family, the human epithelial anion transporter Scl26a6, corresponding to Glu-427 in Sul1 and Glu-443 in Sul2, abolished its ability to perform coupled transport of Cl^−^/HCO_3_^−^ without eliminating its ability to conduct uncoupled anionic current (47). This underscores not only the importance of this conserved residue in transport but also that proper mutagenesis can uncouple the transport function from other functions that a transporter may have.

In this work, we have identified Glu-427 in Sul1 and Glu-443 in Sul2 as only required for transport and not for signaling. We have previously obtained a similar result upon mutagenesis of Asp-358 in the Pho84 transceptor, which also abolished transport with little effect on signaling (43). Hence, transceptor signaling now provides a new useful read-out to identify residues likely to be involved in the binding of the co-transported ion during its passage through a transporter (60). Although there is only indirect evidence at present that such residues are important because they are part of the H^+^-translocation pathway, their strong conservation suggests that Sul1 and Sul2 transport sulfate by a mechanism similar, if not equivalent, to the mechanism proposed for the fungal phosphate transporter, PiPT, and for the human POT peptide transporter (61, 62). In accordance with the transport models proposed in these papers, we suggest that the H^+^-binding site Asp-124 in Sul1 and Asp-140 in Sul2 faces the extracellular side of the plasma membrane and that in the outward open conformation, protonation of this residue is required to abolish repulsion of the negatively charged sulfate molecule so that it can bind to the transporter. This would explain why the residue is essential both for sulfate transport and signaling. Sulfate binding would then trigger a subsequent series of conformational changes, which is associated with its translocation through the passageway of the transporter, one of which may trigger signaling to the PKA pathway. After the latter event has taken place, the H^+^ might be transferred to the next H^+^-binding site, Glu-427 in Sul1 and Glu-443 in Sul2. This second putative H^+^-binding site is located more toward the intracellular face of the transmembrane domain, and its protonation could take place when the transporter adopts the inward open conformation. Such a sequence of events would explain why mutagenesis of Asp-124/Asp-140 abolishes both transport and signaling, whereas the mutagenesis of Glu-427/Glu-443 abolishes only transport and not signaling. In other words, whereas transport requires a complete alternation of outward- and inward-facing conformers of Sul1/2, for signaling, the completion of the transport cycle is not essential. What seems to be necessary for signaling is a specific intermediate transporter conformation, the acquisition of which is not hindered by mutations in Glu-427/Glu-443, which, however, are essential for subsequent steps necessary for completing the transport cycle.

Homology threading (see the Bioinformatics Toolkit Web site) with known H^+^ co-transporters reveals that Sul1,2 show structural similarity with UraA, the major uracil transporter of *E. coli*, which is believed to function as a H^+^-symporter (46). UraA is a member of the so-called nucleobase ascorbate transporter family, also known as NCS2, some bacterial and fungal members of which have been extensively studied in respect to structure-function relationships (63, 64). Such studies have identified residues critical for substrate or H^+^ binding and symport (64, 65). Mutational studies in UraA and homologous transporters, such as the XanQ xanthine transporter of *E. coli* (66) and the UapA uric acid-xanthine transporter of *A. nidulans* (65), have suggested that His-245 is involved in proton binding. His-245 in UraA corresponds to Glu-406 in Sul1 or Glu-422 in Sul2, which are fully conserved in Sul-like transporters and may thus function as major H^+^-binding sites.

##### Endocytosis of Sul1 and Sul2 Is Independent of Signaling

The absence of Sul1 and Sul2 endocytosis after the addition of d-glucosamine 2-sulfate (non-transported signaling agonist) or upon readdition of sulfate to the signaling, non-transporting mutants Sul1^E427Q^ and Sul2^E443Q^ has demonstrated that signaling is not necessarily associated with transceptor endocytosis. The apparent requirement of transport for endocytosis can be explained in different ways. First, an intracellular effect caused by the accumulation of sulfate and/or any of its derived metabolites might be the trigger for endocytosis. This mechanism is not supported by the observation that methionine, a preferred sulfur source for cells, can induce rapid down-regulation of *SUL1,2* transcript levels but does not trigger endocytosis of Sul proteins already present in the plasma membrane (25). Although not contradicting a role for intracellular sulfate itself, it dismisses a role for increased sulfur content in triggering endocytosis of sulfate transporters. Second, the ability of the substrate to elicit in the wild type transporter a specific transient conformation between the signaling conformation and the conformation, which releases sulfate into the cell, may act as initial trigger. Such a conformational change induced by substrate binding may be transmitted to a conformation-sensing domain (loop interaction domain or LID) that regulates site-specific ubiquitination, as recently shown in the case of the Fur4 uracil permease (67). Several examples have been reported of mutant transporters that were deficient in transport and in endocytosis, strongly suggesting a link between the two phenomena. The Gap1^A297V^ mutant lacked the ability to transport citrulline and arginine and failed to become internalized in the presence of citrulline and arginine, despite its ability to bind these substrates (48). This shows the importance of the transporter being able to complete the substrate transport cycle for endocytosis to be triggered. Similar evidence has also been found in other nutrient transporters, such as Smf1, Ftr1, and UapA (49, 50, 52). This is consistent with our observation that Sul1^E427Q^ and Sul2^E443Q^ are defective both in transport and endocytosis, although not in signaling. Hence, a conformation occurring after the signaling event, either before or concomitantly with the intracellular release of sulfate, may act as trigger.

Our work with the Sul1^E427Q^ and Sul2^E443Q^ alleles indicates that the conformational changes involved in signaling are independent of those involved in triggering endocytosis. These results are consistent with our previous results obtained for the Gap1 amino acid transceptor, in which transport, signaling, endocytosis, and even oligoubiquitination could be uncoupled from each other to a large extent, suggesting that different downstream processes are triggered by different conformations of the protein, for instance those occurring sequentially during the transport cycle (68).

##### The Sulfate Transceptors Sul1 and Sul2 Are Tightly Regulated at Multiple Levels upon Sulfate Sensing

Similar to classical receptors and transporters, also transceptors are tightly regulated according to environmental cues. Expression of both *SUL1* and *SUL2* is up-regulated such that Sul1 and Sul2 increase their level at the plasma membrane upon sulfate scarcity (21). We have also shown here that their transport capacity and expression level drop rapidly upon the readdition of sulfate, a process that occurs concomitantly with substrate-induced endocytosis and vacuolar degradation. It has to be noted that the down-regulation of transport capacity occurred much faster than the rate of endocytosis, suggesting that an additional level of regulation might inhibit transport even before Sul1 and Sul2 are removed from the plasma membrane through endocytosis. Such an additional inhibition of transport activity in the absence of endocytosis has been reported for the Gap1 amino acid transceptor (69) and has also been suggested in the case of Sul2 (25). This rapid regulation could, for example, consist of an allosteric inhibition that changes the conformation of the transporter, reducing the transport capacity upon extracellular increase in sulfate.

Such a mechanism for rapid down-regulation of the Sul1 and Sul2 transceptors could be important not only to lower sulfate uptake activity but also to limit transceptor signaling to the PKA pathway, so as to prevent its overactivation, similar to what is well known for classical receptors (70). In this respect, it is interesting to note that the long term regulatory response of the *HSP12* PKA target in cases where signaling took place without transport (use of d-glucosamine 2-sulfate or of the Glu to Gln mutant transporters in putative proton binding sites) is attenuated compared with that with sulfate in wild type cells. This suggests that transceptor signaling is especially important for the rapid response and that a second mechanism based on intracellular sulfate sensing may be responsible for long term maintenance of the effect on the PKA targets.

## Footnotes

2 H. N. Kankipati, M. Rubio-Texeira, D. Castermans, G. Diallinas, and J. M. Thevelein, unpublished results.

## Footnotes

3 The abbreviation used is:

TMD: transmembrane domain.

## References

[1] Conrad M. Schothorst J. Kankipati H. N. Van Zeebroeck G. Rubio-Texeira M. Thevelein J. M. (2014) Nutrient sensing and signaling in the yeast *Saccharomyces cerevisiae*. FEMS Microbiol. Rev. 38, 254–299 24483210 10.1111/1574-6976.12065 PMC4238866

[2] Holsbeeks I. Lagatie O. Van Nuland A. Van de Velde S. Thevelein J. M. (2004) The eukaryotic plasma membrane as a nutrient-sensing device. Trends Biochem. Sci. 29, 556–564 15450611 10.1016/j.tibs.2004.08.010

[3] Thevelein J. M. Voordeckers K. (2009) Functioning and evolutionary significance of nutrient transceptors. Mol. Biol. Evol. 26, 2407–2414 19651853 10.1093/molbev/msp168

[4] Donaton M. C. Holsbeeks I. Lagatie O. Van Zeebroeck G. Crauwels M. Winderickx J. Thevelein J. M. (2003) The Gap1 general amino acid permease acts as an amino acid sensor for activation of protein kinase A targets in the yeast *Saccharomyces cerevisiae*. Mol. Microbiol. 50, 911–929 14617151 10.1046/j.1365-2958.2003.03732.x

[5] Van Zeebroeck G. Bonini B. M. Versele M. Thevelein J. M. (2009) Transport and signaling via the amino acid binding site of the yeast Gap1 amino acid transceptor. Nat. Chem. Biol. 5, 45–52 19060912 10.1038/nchembio.132

[6] Giots F. Donaton M. C. Thevelein J. M. (2003) Inorganic phosphate is sensed by specific phosphate carriers and acts in concert with glucose as a nutrient signal for activation of the protein kinase A pathway in the yeast *Saccharomyces cerevisiae*. Mol. Microbiol. 47, 1163–1181 12581367 10.1046/j.1365-2958.2003.03365.x

[7] Popova Y. Thayumanavan P. Lonati E. Agrochão M. Thevelein J. M. (2010) Transport and signaling through the phosphate-binding site of the yeast Pho84 phosphate transceptor. Proc. Natl. Acad. Sci. U.S.A. 107, 2890–2895 20133652 10.1073/pnas.0906546107 PMC2840322

[8] Van Nuland A. Vandormael P. Donaton M. Alenquer M. Lourenço A. Quintino E. Versele M. Thevelein J. M. (2006) Ammonium permease-based sensing mechanism for rapid ammonium activation of the protein kinase A pathway in yeast. Mol. Microbiol. 59, 1485–1505 16468990 10.1111/j.1365-2958.2005.05043.x

[9] Jauniaux J. C. Grenson M. (1990) GAP1, the general amino acid permease gene of *Saccharomyces cerevisiae*: nucleotide sequence, protein similarity with the other bakers yeast amino acid permeases, and nitrogen catabolite repression. Eur. J. Biochem. 190, 39–44 2194797 10.1111/j.1432-1033.1990.tb15542.x

[10] Lagerstedt J. O. Zvyagilskaya R. Pratt J. R. Pattison-Granberg J. Kruckeberg A. L. Berden J. A. Persson B. L. (2002) Mutagenic and functional analysis of the C-terminus of *Saccharomyces cerevisiae* Pho84 phosphate transporter. FEBS Lett. 526, 31–37 12208499 10.1016/s0014-5793(02)03109-5

[11] Petersson J. Pattison J. Kruckeberg A. L. Berden J. A. Persson B. L. (1999) Intracellular localization of an active green fluorescent protein-tagged Pho84 phosphate permease in *Saccharomyces cerevisiae*. FEBS Lett. 462, 37–42 10580087 10.1016/s0014-5793(99)01471-4

[12] Springael J. Y. André B. (1998) Nitrogen-regulated ubiquitination of the Gap1 permease of *Saccharomyces cerevisiae*. Mol. Biol. Cell 9, 1253–1263 9614172 10.1091/mbc.9.6.1253 PMC25348

[13] Stanbrough M. Magasanik B. (1995) Transcriptional and posttranslational regulation of the general amino acid permease of *Saccharomyces cerevisiae*. J. Bacteriol. 177, 94–102 7798155 10.1128/jb.177.1.94-102.1995 PMC176561

[14] Thevelein J. M. de Winde J. H. (1999) Novel sensing mechanisms and targets for the cAMP-protein kinase A pathway in the yeast *Saccharomyces cerevisiae*. Mol. Microbiol. 33, 904–918 10476026 10.1046/j.1365-2958.1999.01538.x

[15] Hundal H. S. Taylor P. M. (2009) Amino acid transceptors: gate keepers of nutrient exchange and regulators of nutrient signaling. Am. J. Physiol. Endocrinol. Metab. 296, E603–E613 19158318 10.1152/ajpendo.91002.2008 PMC2670634

[16] Pérez-Torras S. Vidal-Pla A. Cano-Soldado P. Huber-Ruano I. Mazo A. Pastor-Anglada M. (2013) Concentrative nucleoside transporter 1 (hCNT1) promotes phenotypic changes relevant to tumor biology in a translocation-independent manner. Cell Death Dis. 4, e648 23722537 10.1038/cddis.2013.173 PMC3674379

[17] Walch-Liu P. Forde B. G. (2008) Nitrate signalling mediated by the *NRT1.1* nitrate transporter antagonises l-glutamate-induced changes in root architecture. Plant J. 54, 820–828 18266918 10.1111/j.1365-313X.2008.03443.x

[18] Hyde R. Cwiklinski E. L. MacAulay K. Taylor P. M. Hundal H. S. (2007) Distinct sensor pathways in the hierarchical control of SNAT2, a putative amino acid transceptor, by amino acid availability. J. Biol. Chem. 282, 19788–19798 17488712 10.1074/jbc.M611520200

[19] Zhang B. Pasini R. Dan H. Joshi N. Zhao Y. Leustek T. Zheng Z. L. (2014) Aberrant gene expression in the *Arabidopsis* SULTR1;2 mutants suggests a possible regulatory role for this sulfate transporter in response to sulfur nutrient status. Plant J. 77, 185–197 24308460 10.1111/tpj.12376

[20] Breton A. Surdin-Kerjan Y. (1977) Sulfate uptake in *Saccharomyces cerevisiae*: biochemical and genetic study. J. Bacteriol. 132, 224–232 199574 10.1128/jb.132.1.224-232.1977 PMC221848

[21] Cherest H. Davidian J. C. Thomas D. Benes V. Ansorge W. Surdin-Kerjan Y. (1997) Molecular characterization of two high affinity sulfate transporters in *Saccharomyces cerevisiae*. Genetics 145, 627–635 9055073 10.1093/genetics/145.3.627 PMC1207848

[22] Takahashi H. Buchner P. Yoshimoto N. Hawkesford M. J. Shiu S. H. (2011) Evolutionary relationships and functional diversity of plant sulfate transporters. Front. Plant Sci. 2, 119 22629272 10.3389/fpls.2011.00119 PMC3355512

[23] Marzluf G. A. (1997) Molecular genetics of sulfur assimilation in filamentous fungi and yeast. Annu. Rev. Microbiol. 51, 73–96 9343344 10.1146/annurev.micro.51.1.73

[24] Boer V. M. de Winde J. H. Pronk J. T. Piper M. D. (2003) The genome-wide transcriptional responses of *Saccharomyces cerevisiae* grown on glucose in aerobic chemostat cultures limited for carbon, nitrogen, phosphorus, or sulfur. J. Biol. Chem. 278, 3265–3274 12414795 10.1074/jbc.M209759200

[25] Jennings M. L. Cui J. (2012) Inactivation of *Saccharomyces cerevisiae* sulfate transporter Sul2p: use it and lose it. Biophys. J. 102, 768–776 22385847 10.1016/j.bpj.2012.01.005 PMC3283770

[26] Lillie S. H. Pringle J. R. (1980) Reserve carbohydrate metabolism in *Saccharomyces cerevisiae*: responses to nutrient limitation. J. Bacteriol. 143, 1384–1394 6997270 10.1128/jb.143.3.1384-1394.1980 PMC294518

[27] Hirimburegama K. Durnez P. Keleman J. Oris E. Vergauwen R. Mergelsberg H. Thevelein J. M. (1992) Nutrient-induced activation of trehalase in nutrient-starved cells of the yeast *Saccharomyces cerevisiae*: cAMP is not involved as second messenger. J. Gen. Microbiol. 138, 2035–2043 1336029 10.1099/00221287-138-10-2035

[28] Schepers W. Van Zeebroeck G. Pinkse M. Verhaert P. Thevelein J. M. (2012) *In vivo* phosphorylation of Ser^21^ and Ser^83^ during nutrient-induced activation of the yeast protein kinase A (PKA) target trehalase. J. Biol. Chem. 287, 44130–44142 23155055 10.1074/jbc.M112.421503 PMC3531729

[29] Thevelein J. M. Beullens M. (1985) Cyclic AMP and the stimulation of trehalase activity in the yeast Saccharomyces cerevisiae by carbon sources, nitrogen sources and inhibitors of protein synthesis. J. Gen. Microbiol. 131, 3199–3209 3007655 10.1099/00221287-131-12-3199

[30] Cherest H. Surdin-Kerjan Y. (1992) Genetic analysis of a new mutation conferring cysteine auxotrophy in *Saccharomyces cerevisiae*: updating of the sulfur metabolism pathway. Genetics 130, 51–58 1732168 10.1093/genetics/130.1.51 PMC1204804

[31] Sikorski R. S. Hieter P. (1989) A system of shuttle vectors and yeast host strains designed for efficient manipulation of DNA in *Saccharomyces cerevisiae*. Genetics 122, 19–27 2659436 10.1093/genetics/122.1.19 PMC1203683

[32] Yanisch-Perron C. Vieira J. Messing J. (1985) Improved M13 phage cloning vectors and host strains: nucleotide sequences of the M13mp18 and pUC19 vectors. Gene 33, 103–119 2985470 10.1016/0378-1119(85)90120-9

[33] Lowry O. H. Rosebrough N. J. Farr A. L. Randall R. J. (1951) Protein measurement with the Folin phenol reagent. J. Biol. Chem. 193, 265–275 14907713

[34] Colombo S. Ma P. Cauwenberg L. Winderickx J. Crauwels M. Teunissen A. Nauwelaers D. de Winde J. H. Gorwa M. F. Colavizza D. Thevelein J. M. (1998) Involvement of distinct G-proteins, Gpa2 and Ras, in glucose- and intracellular acidification-induced cAMP signalling in the yeast *Saccharomyces cerevisiae*. EMBO J. 17, 3326–3341 9628870 10.1093/emboj/17.12.3326 PMC1170671

[35] Castermans D. Somers I. Kriel J. Louwet W. Wera S. Versele M. Janssens V. Thevelein J. M. (2012) Glucose-induced posttranslational activation of protein phosphatases *PP2A* and *PP1* in yeast. Cell Res. 22, 1058–1077 22290422 10.1038/cr.2012.20 PMC3367521

[36] Rubio-Texeira M. Van Zeebroeck G. Thevelein J. M. (2012) Peptides induce persistent signaling from endosomes by a nutrient transceptor. Nat. Chem. Biol. 8, 400–408 22388927 10.1038/nchembio.910

[37] Rubio-Texeira M. Kaiser C. A. (2006) Amino acids regulate retrieval of the yeast general amino acid permease from the vacuolar targeting pathway. Mol. Biol. Cell 17, 3031–3050 16641373 10.1091/mbc.E05-07-0669 PMC1483039

[38] Adams A. Gottschling D. Kaiser C. (1996) Methods in Yeast Genetics: A Laboratory Course Manual, Cold Spring Harbor Laboratory Press, Cold Spring Harbor, NY

[39] Hall C. Brachat S. Dietrich F. S. (2005) Contribution of horizontal gene transfer to the evolution of *Saccharomyces cerevisiae*. Eukaryot. Cell 4, 1102–1115 15947202 10.1128/EC.4.6.1102-1115.2005 PMC1151995

[40] Roomans G. M. Kuypers G. A. Theuvenet A. P. Borst-Pauwels G. W. (1979) Kinetics of sulfate uptake by yeast. Biochim. Biophys. Acta 551, 197–206 34436 10.1016/0005-2736(79)90365-1

[41] Garczarek F. Wang J. El-Sayed M. A. Gerwert K. (2004) The assignment of the different infrared continuum absorbance changes observed in the 3000–1800-cm^−1^ region during the bacteriorhodopsin photocycle. Biophys. J. 87, 2676–2682 15298873 10.1529/biophysj.104.046433 PMC1304686

[42] Sahin-Tóth M. Karlin A. Kaback H. R. (2000) Unraveling the mechanism of the lactose permease of *Escherichia coli*. Proc. Natl. Acad. Sci. U.S.A. 97, 10729–10732 10984523 10.1073/pnas.200351797 PMC27091

[43] Samyn D. R. Ruiz-Pávon L. Andersson M. R. Popova Y. Thevelein J. M. Persson B. L. (2012) Mutational analysis of putative phosphate- and proton-binding sites in the *Saccharomyces cerevisiae* Pho84 phosphate:H^+^ transceptor and its effect on signalling to the PKA and PHO pathways. Biochem. J. 445, 413–422 22587366 10.1042/BJ20112086

[44] Smirnova I. N. Kasho V. Kaback H. R. (2008) Protonation and sugar binding to LacY. Proc. Natl. Acad. Sci. U.S.A. 105, 8896–8901 18567672 10.1073/pnas.0803577105 PMC3021437

[45] Käll L. Krogh A. Sonnhammer E. L. (2005) An HMM posterior decoder for sequence feature prediction that includes homology information. Bioinformatics 21, i251–i257 15961464 10.1093/bioinformatics/bti1014

[46] Lu F. Li S. Jiang Y. Jiang J. Fan H. Lu G. Deng D. Dang S. Zhang X. Wang J. Yan N. (2011) Structure and mechanism of the uracil transporter UraA. Nature 472, 243–246 21423164 10.1038/nature09885

[47] Ohana E. Shcheynikov N. Yang D. So I. Muallem S. (2011) Determinants of coupled transport and uncoupled current by the electrogenic SLC26 transporters. J. Gen. Physiol. 137, 239–251 21282402 10.1085/jgp.201010531 PMC3032377

[48] Cain N. E. Kaiser C. A. (2011) Transport activity-dependent intracellular sorting of the yeast general amino acid permease. Mol. Biol. Cell 22, 1919–1929 21471002 10.1091/mbc.E10-10-0800 PMC3103407

[49] Felice M. R. De Domenico I. Li L. Ward D. M. Bartok B. Musci G. Kaplan J. (2005) Post-transcriptional regulation of the yeast high affinity iron transport system. J. Biol. Chem. 280, 22181–22190 15817488 10.1074/jbc.M414663200

[50] Gournas C. Amillis S. Vlanti A. Diallinas G. (2010) Transport-dependent endocytosis and turnover of a uric acid-xanthine permease. Mol. Microbiol. 75, 246–260 20002879 10.1111/j.1365-2958.2009.06997.x

[51] Jensen L. T. Carroll M. C. Hall M. D. Harvey C. J. Beese S. E. Culotta V. C. (2009) Down-regulation of a manganese transporter in the face of metal toxicity. Mol. Biol. Cell 20, 2810–2819 19369420 10.1091/mbc.E08-10-1084 PMC2695789

[52] Liu X. F. Culotta V. C. (1999) Mutational analysis of *Saccharomyces cerevisiae* Smf1p, a member of the Nramp family of metal transporters. J. Mol. Biol. 289, 885–891 10369769 10.1006/jmbi.1999.2815

[53] Séron K. Blondel M. O. Haguenauer-Tsapis R. Volland C. (1999) Uracil-induced down-regulation of the yeast uracil permease. J. Bacteriol. 181, 1793–1800 10074071 10.1128/jb.181.6.1793-1800.1999 PMC93577

[54] Diallinas G. (2013) Allopurinol and xanthine use different translocation mechanisms and trajectories in the fungal UapA transporter. Biochimie 95, 1755–1764 23791789 10.1016/j.biochi.2013.05.013

[55] Jiang X. McDermott J. R. Ajees A. A. Rosen B. P. Liu Z. (2010) Trivalent arsenicals and glucose use different translocation pathways in mammalian GLUT1. Metallomics 2, 211–219 21069159 10.1039/b920471g PMC3733330

[56] Didion T. Regenberg B. Jørgensen M. U. Kielland-Brandt M. C. Andersen H. A. (1998) The permease homologue Ssy1p controls the expression of amino acid and peptide transporter genes in *Saccharomyces cerevisiae*. Mol. Microbiol. 27, 643–650 9489675 10.1046/j.1365-2958.1998.00714.x

[57] Iraqui I. Vissers S. Bernard F. de Craene J. O. Boles E. Urrestarazu A. André B. (1999) Amino acid signaling in *Saccharomyces cerevisiae*: a permease-like sensor of external amino acids and F-Box protein Grr1p are required for transcriptional induction of the *AGP1* gene, which encodes a broad-specificity amino acid permease. Mol. Cell. Biol. 19, 989–1001 9891035 10.1128/mcb.19.2.989 PMC116030

[58] Klasson H. Fink G. R. Ljungdahl P. O. (1999) Ssy1p and Ptr3p are plasma membrane components of a yeast system that senses extracellular amino acids. Mol. Cell. Biol. 19, 5405–5416 10409731 10.1128/mcb.19.8.5405 PMC84383

[59] Ozcan S. Dover J. Johnston M. (1998) Glucose sensing and signaling by two glucose receptors in the yeast *Saccharomyces cerevisiae*. EMBO J. 17, 2566–2573 9564039 10.1093/emboj/17.9.2566 PMC1170598

[60] Schothorst J. Kankipati H. N. Conrad M. Samyn D. R. Van Zeebroeck G. Popova Y. Rubio-Texeira M. Persson B. L. Thevelein J. M. (2013) Yeast nutrient transceptors provide novel insight in the functionality of membrane transporters. Curr. Genet. 59, 197–206 24114446 10.1007/s00294-013-0413-y PMC3824880

[61] Doki S. Kato H. E. Solcan N. Iwaki M. Koyama M. Hattori M. Iwase N. Tsukazaki T. Sugita Y. Kandori H. Newstead S. Ishitani R. Nureki O. (2013) Structural basis for dynamic mechanism of proton-coupled symport by the peptide transporter POT. Proc. Natl. Acad. Sci. U.S.A. 110, 11343–11348 23798427 10.1073/pnas.1301079110 PMC3710879

[62] Pedersen B. P. Kumar H. Waight A. B. Risenmay A. J. Roe-Zurz Z. Chau B. H. Schlessinger A. Bonomi M. Harries W. Sali A. Johri A. K. Stroud R. M. (2013) Crystal structure of a eukaryotic phosphate transporter. Nature 496, 533–536 23542591 10.1038/nature12042 PMC3678552

[63] Diallinas G. Gournas C. (2008) Structure-function relationships in the nucleobase-ascorbate transporter (NAT) family: lessons from model microbial genetic systems. Channels 2, 363–372 18981714 10.4161/chan.2.5.6902

[64] Frillingos S. (2012) Insights to the evolution of nucleobase-ascorbate transporters (NAT/NCS2 family) from the Cys-scanning analysis of xanthine permease XanQ. Int. J. Biochem. Mol. Biol. 3, 250–272 23097742 PMC3476789

[65] Kosti V. Lambrinidis G. Myrianthopoulos V. Diallinas G. Mikros E. (2012) Identification of the substrate recognition and transport pathway in a eukaryotic member of the nucleobase-ascorbate transporter (NAT) family. PLoS One 7, e41939 22848666 10.1371/journal.pone.0041939 PMC3405029

[66] Mermelekas G. Georgopoulou E. Kallis A. Botou M. Vlantos V. Frillingos S. (2010) Cysteine-scanning analysis of helices TM8, TM9a, and TM9b and intervening loops in the YgfO xanthine permease: a carboxyl group is essential at Asp-276. J. Biol. Chem. 285, 35011–35020 20802252 10.1074/jbc.M110.170415 PMC2966115

[67] Keener J. M. Babst M. (2013) Quality control and substrate-dependent downregulation of the nutrient transporter Fur4. Traffic 14, 412–427 23305501 10.1111/tra.12039 PMC3594327

[68] Van Zeebroeck G. Rubio-Texeira M. Schothorst J. Thevelein J. M. (2014) Specific analogues uncouple transport, signalling, oligo-ubiquitination and endocytosis in the yeast Gap1 amino acid transceptor. Mol. Microbiol. 93, 213–233 24852066 10.1111/mmi.12654 PMC4285233

[69] Risinger A. L. Cain N. E. Chen E. J. Kaiser C. A. (2006) Activity-dependent reversible inactivation of the general amino acid permease. Mol. Biol. Cell 17, 4411–4419 16885415 10.1091/mbc.E06-06-0506 PMC1635348

[70] Sorkin A. Von Zastrow M. (2002) Signal transduction and endocytosis: close encounters of many kinds. Nat. Rev. Mol. Cell Biol. 3, 600–614 12154371 10.1038/nrm883

